# HEALTH AND FUNCTION FROM MID LIFE TO LATE ADULTHOOD: FINDINGS FROM THE STUDY OF WOMEN’S HEALTH ACROSS THE NATION (SWAN)

**DOI:** 10.1093/geroni/igad104.2029

**Published:** 2023-12-21

**Authors:** Carrie Karvonen-Gutierrez, Nancy Fugate Woods

## Abstract

Midlife (age 40-64) is characterized by physiologic, psychosocial and situational change, and a time when women experience the seminal endocrinological event of menopause. From a lifecourse perspective, understanding the relationship between midlife and older adult health is critical, particularly for identifying modifiable factors in midlife that impact later life health and functioning. Previous research has demonstrated that midlife health and functioning is dynamic and thus potentially amenable to intervention, though there remain crucial gaps in our understanding of this period. The Study of Women’s Health Across the Nation (SWAN) is an observational multi-racial/ethnic (White, Black, Chinese, Japanese, Hispanic) longitudinal study of 3,302 women recruited in 1996 from 7 sites in the United States (Boston, MA; Newark, NJ; Pittsburgh, PA; Detroit-area, MI; Chicago, IL; Oakland, CA; Los Angeles, CA). Over the past 27 years, women have participated in up to 17 near-annual study visits, spanning from midlife (age 42-52 years at baseline) to later life (age 69-79 years). This symposium features new science from SWAN including: an examination of rate of change in depressive symptoms across the menopausal transition (Avis); the impact of impaired glucose levels in midlife on muscle quality and power (Leis); the importance of sleep regularity for cognitive function (Derby); and the association of urinary incontinence with disability (Dugan). The symposium provides information regarding data and biospecimen access for investigators interested in using SWAN resources (Brooks). The Discussant will consider the implications of studying midlife health and function in relation to health and well-being in late adulthood.

